# Evaluation of small vessel disease burden on MRI and stroke outcomes

**DOI:** 10.3389/fneur.2025.1628787

**Published:** 2025-07-09

**Authors:** Janet Nguyen, Nicholas Vo, Peter D. Chang, Chanon Chantaduly, Wengui Yu, Jennifer E. Soun

**Affiliations:** ^1^School of Medicine, University of California, Irvine, Irvine, CA, United States; ^2^Department of Radiological Sciences, University of California, Irvine, Orange, CA, United States; ^3^California University of Science and Medicine, Colton, CA, United States; ^4^Center for Artificial Intelligence in Diagnostic Medicine, University of California, Irvine, Irvine, CA, United States; ^5^Department of Neurology, University of California, Irvine, Orange, CA, United States

**Keywords:** small vessel disease, MRI, stroke, white matter hyperintensities, lacunar infarcts, perivascular spaces, cerebral microbleeds

## Abstract

**Purpose:**

We aimed to assess whether a composite SVD score derived from MRI features improves stroke outcome prediction when integrated with clinical factors.

**Materials and methods:**

A 2019–2022 retrospective analysis included patients who had MRI prior to stroke admission. A semi-automated approach evaluated SVD MRI markers (white matter hyperintensities (WMH), lacunar infarcts, perivascular spaces (PVS), and cerebral microbleeds (CMBs)) using continuous and categorical measures to create a composite SVD score. Multivariate regression analyses compared performance across three models: (1) SVD score, (2) clinical factors, and (3) SVD score + clinical factors for outcomes, including stroke etiology, ICU and hospital stay, NIH Stroke Scale (NIHSS), 90 day modified Rankin Scale (mRS), functional independence (mRS < 2), and stroke recurrence.

**Results:**

Forty-eight patients were included in this study. The combined SVD + clinical factors model outperformed other models in predicting functional independence with area under the curve (AUC) 0.58 (95% CI 0.41–0.75) and stroke etiologies of large artery atherosclerosis AUC 0.78 (0.62–0.91), small vessel occlusion 0.65 (0.41–0.88), and other determined etiology 0.74 (0.37–0.96). The combined model also better predicted the following outcomes with lower mean absolute error (MAE): NIHSS MAE 5.16 (3.80–6.69), ICU days 1.26 (0.86–1.66), total length of stay 2.62 (2.08–3.17), and 90 day mRS 1.74 (1.39–2.12).

**Conclusion:**

Combining an SVD score with clinical variables improved prediction of stroke outcomes when compared to either predictor alone, although the added value is modest and requires further validation. Nonetheless, integrating an SVD score into clinical practice may guide management in acute stroke settings and support risk stratification and prognostication.

## 1 Introduction

Cerebral small vessel disease (SVD) which affects small blood vessels in the brain contributes to increased risk of stroke and dementia (1). SVD can be evaluated noninvasively on MRI. Characteristic neuroimaging markers of SVD include white matter hyperintensities (WMH), lacunar infarcts, perivascular spaces (PVS), and cerebral microbleeds (CMBs) visible on fluid-attenuated inversion recovery (FLAIR), T2-weighted, and susceptibility-weighted imaging (SWI) MRI sequences, respectively (2).

These neuroimaging markers have been found to correlate to stroke risk and outcome. WMHs have been linked to increased stroke incidence and increased risk of recurrent stroke based on WMH severity (3–5). Lacunar infarcts, smaller strokes that affect deep gray and white matter structures, are associated with an increased risk of recurrent stroke, cognitive decline, and dementia (6). Patients with an increased burden of small-size PVS have been shown to have increased vascular events such as myocardial infarction, stroke, and vascular-related death (7). Additionally, PVS located in the basal ganglia have been correlated to the other SVD components and to increased future ischemic stroke risk in patients with prior ischemic stroke or transient ischemic attack (TIA) (8). A higher burden of CMBs has also been linked to increased stroke risk in a prospective study of the general population (9).

Prior studies have evaluated the overall SVD burden by calculating an SVD score based on a combination of these imaging markers, showing promise in predicting stroke outcomes (10–12). However, current methods present several important limitations. First, the categorical nature of the score reduces granularity, potentially obscuring clinically meaningful differences in lesion burden. For example, researchers have found that the number of CMBs on a continuous scale and the presence of lacunes as a binary output were independent predictors of functional stroke outcome measured by the modified Rankin scale (mRS), supporting the idea that clinical SVD features may be better assessed using a hybrid approach of continuous and categorical metrics (13). Second, although SVD scores have shown a significant association with stroke, cognitive decline, stroke-related mortality, and recurrent stroke, the score assumes equal weighting of each marker, although different features may have distinct prognostic relevance (11, 14). Third, inter-rater variability may affect reliability, particularly when qualitative thresholds are used. Lastly, despite the evidence supporting SVD score as a valid assessment of stroke-related outcomes, manual calculation of the components of the SVD score can be time-consuming and error-prone, limiting their clinical utility (15, 16). Recent advances in artificial intelligence (AI) and machine learning (ML) have accelerated efforts to automate neuroimaging analysis; however, the utility of automated tools to evaluate SVD burden remains underexplored. Thus, improvements are needed to calculate the SVD score in a timely and accurate fashion.

To address these limitations, we developed a semi-automated method to detect all four components of the SVD score and evaluated SVD imaging markers using both continuous and categorical assessments in a composite SVD score. The purpose of our study was to determine whether a composite SVD score could provide additional benefit in comparison to clinical risk factors to predict stroke etiologies and outcomes in stroke patients. Specifically, we compared three multivariate models: (1) SVD score alone, (2) clinical factors alone, and (3) combined SVD score + clinical factors.

## 2 Materials and methods

### 2.1 Patient selection

This study was a retrospective analysis of patients admitted between 2019 and 2022 for stroke to the University of California, Irvine (UCI), a comprehensive stroke center. The study selected only patients who had received a brain MRI prior to their stroke admission. Patient demographics (age, sex, race, ethnicity), stroke risk factors (hypertension, diabetes mellitus, hyperlipidemia, atrial fibrillation, heart failure, obesity, and smoking history), stroke etiologies (large artery atherosclerosis, cardioembolism, small vessel occlusion, stroke of other determined etiology, and stroke of undetermined etiology), and clinical outcomes (total hospital length of stay, total ICU days, NIHSS on stroke admission, 90 day mRS, 90 day functional independence [binary mRS], and stroke recurrence were recorded). 90 day functional independence was a binary output determined by an mRS cutoff of 2, where mRS < 2 was considered functionally independent. Stroke recurrence was determined by stroke readmission during the study period. The study was conducted in accordance with local Institutional Review Board protocols and informed consent was waived.

### 2.2 MRI acquisition and semi-automated processing

All MRIs were obtained on either a 1.5 T or 3 T scanner (Siemens Avanto 1.5 T, Siemens Trio 3 T, Siemens Vida 3 T, or Philips Achieva 3 T). Only MRIs with axial FLAIR (3–7 mm slice thickness), axial T2 (3–7 mm), and axial SWI (2 mm) sequences were included. If necessary, the MRI sequences were pre-processed by padding into a square shape before being uploaded into a dedicated in-house viewer for analysis.

A simple threshold-based semi-automated segmentation strategy was implemented for SVD marker annotation. First, the user manually selected a representative area within the target region on a single image slice. Subsequently, an intensity threshold was set based on the voxel statistics within the user-defined region, calculated as either a lower intensity threshold (80% of mean within selected region) for hyperintense targets (perivascular spaces, lacunes) or an upper intensity threshold (120% of mean within selected region) for hypointense targets (microbleeds). The user then manually selected all target regions on a slice-by-slice basis, with the option to repeat the process to redefine thresholds for different lesions as required.

### 2.3 SVD analysis

**Figure 1:**
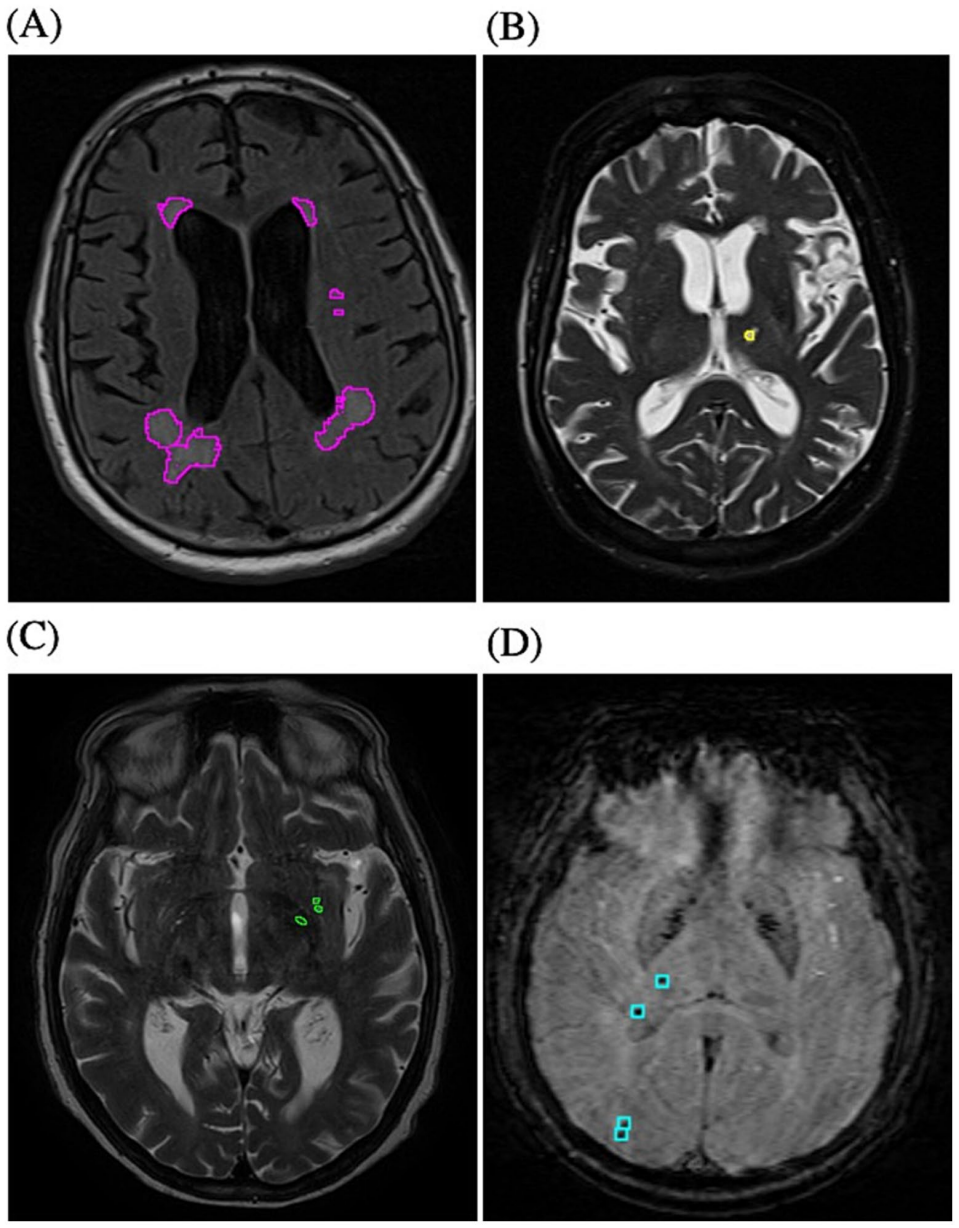
Semi-automated segmentation of SVD markers on MRI, including white matter hyperintensities seen on FLAIR (**A**, purple), lacunar infarcts on T2 (**B**, yellow), perivascular spaces on T2 (**C**, green), and cerebral microbleeds on SWI (**D**, blue).

MRIs were screened for SVD markers in accordance with criteria set forth by the Standards for Reporting Vascular Changes on Neuroimaging (STRIVE) (12). SVD analysis using both continuous and categorical assessments was performed by two readers, 1 board-certified neuroradiologist with 8 years experience and either of 2 medical students (Figure 1). The neuroradiologist provided consensus reading for all cases reviewed by the other readers. SVD categorical assessment was performed based on a prior established SVD score (10).

WMH in the deep and periventricular supratentorial white matter on FLAIR sequence were segmented for total volume (mm (3)) in continuous variable evaluation and for Fazekas grading (0–3) in categorical evaluation (17). Lacunes were defined as round or ovoid, fluid-filled, CSF signal intensity foci within a diameter of 3–15 mm and located in the deep grey or white matter structures or brainstem. Lacunes were segmented on T2 for total number of lesions. PVS were classified as small fluid-filled CSF signal intensity spaces <3 mm typically coursing along perforating vessels. PVS were segmented at the level of the basal ganglia on T2 choosing the slice with the most number of PVS on one side. The total number of lesions based on this definition and grading based on a validated semi-quantitative scale were recorded for continuous and categorical assessment, respectively (10, 18). CMBs were defined as small (approximately 2–5 mm), low signal intensity foci on SWI. Total number of CMBs was recorded.

For continuous assessment, the total volume was analyzed for WMH, and total number of lesions was analyzed for lacunes, PVS, and CMBs. For the categorical evaluation, an SVD summation score from 0 to 4 was evaluated based on the following criteria: (1) confluent WMH were given 1 point (deep WMH Fazekas grade 2–3 or periventricular WMH Fazekas grade 3), (2) presence of lacunes was given 1 point, (3) PVS grades 2–4 (at least 11 PVS) was given 1 point, and (3) presence of CMBs was given 1 point. A composite SVD score was created which included continuous and categorical assessments for each imaging marker.

### 2.4 Statistical analysis

The following independent variables were evaluated: both continuous and categorical assessment of WMH, lacunes, PVS, and CMBs, as well as the categorical SVD score. Interrater agreement was assessed for categorical SVD score evaluation using Cohen’s kappa. Clinical variables including patient demographics and stroke risk factors were also evaluated as independent variables. Dependent stroke outcome variables included stroke etiologies, total hospital length of stay, total ICU days, NIHSS on stroke admission, 90 day mRS, 90 day functional independence, and stroke recurrence. Statistical significance was set at *p* < 0.05.

#### 2.4.1 Univariate analysis

For univariate analysis, *t*-tests, Pearson correlation, and Chi-square were performed where appropriate for continuous and categorical assessment. To adjust for multiple comparisons, we also implemented a false discovery rate (FDR) control procedure using the Benjamini-Hochberg method with adjusted *p* values reported.

#### 2.4.2 Multivariate models

Multivariate models were created to predict dependent variable stroke outcomes based on independent variables including continuous and categorical SVD variables as well as clinical factors. Three different combinations of multivariate model inputs were evaluated: (1) composite SVD only, (2) clinical only, and (3) combined SVD + clinical factors (full feature set). Separate models were trained for each target dependent variable stroke outcome. Binary variable outcomes were modeled with multivariate logistic regression, and continuous variable outcomes were modeled with multivariate linear regression.

Prior to multivariate modeling, the top features for each dependent variable were ranked using univariate statistics (unpaired *t*-test, Pearson correlation, Chi-square test). A recursive feature selection technique was used to identify the top N variables needed for optimal performance. A leave-one-out cross-validation technique was implemented for all experiments. All reported results reflect validation set statistics. Binary outcomes were assessed using area under the receiver operating characteristic curve (AUC) as well as overall accuracy, sensitivity, specificity, positive predictive value (PPV), and negative predictive value (NPV) based on an optimal threshold derived from the Youden index. Continuous outcomes were assessed using mean absolute error (MAE). A Wilcoxon signed rank test was used to evaluate the statistical significance of model differences. 95% confidence intervals were determined via bootstrapping with 1,000 repetitions.

## 3 Results

A total of 48 stroke patients who had a baseline MRI were included in our study. Patient characteristics and clinical outcomes are summarized in Table 1, and SVD imaging markers are described in Table 2. There was no missing data for our cohort. Hypertension was excluded from analysis since almost every patient (47/48) had this risk factor. Mean time interval between MRI acquisition and stroke admission was 614.38 days. Stroke recurrence occurred in 9 patients and averaged 218.67 days in the study period. SVD semi-automated segmentation time averaged 5 min. The agreement between readers for categorical SVD score evaluation was high (Cohen’s kappa = 0.85).

**Table 1:** Baseline demographics, stroke etiologies, and clinical outcomes.

**Clinical variables**	**Patients, *N* = 48**
*Baseline demographics*	
Age, years mean (SD)	71.2 (13.5)
Sex, Male%	46.0
Hypertension, %	98.0
Diabetes Mellitus, %	62.5
Hyperlipidemia, %	62.5
Atrial Fibrillation, %	35.4
*Stroke etiology*	
Large Artery atherosclerosis, %	33.33
Cardioembolism, %	33.33
Small vessel occlusion, %	18.75
Stroke of other determined etiology, %	6.25
Stroke of undetermined etiology, %	8.33
*Clinical outcomes*	
Total Length of stay, days mean (SD)	4.0 (3.3)
Total ICU stay, days mean (SD)	3.3 (1.6)
NIHSS on stroke admission, median (IQR)	6 (3–13.3)
90 day mRS, median (IQR)	2 (1–5)
90 day functional independence, %	29.2
Stroke recurrence, *n*	9

**Table 2:** Continuous and categorical SVD imaging markers.

**SVD imaging variables**	**Patients, *N* = 48**
*Continuous SVD assessment*	
WMH volume in mm^3^, mean (SD)	9308.6 (11105.0)
Lacunes, *n* mean (SD)	2.9 (4.3)
PVS, *n* mean (SD)	10.1 (8.3)
CMBs, *n* mean (SD)	5.5 (14.3)
*Categorical SVD assessment*	
WMH Fazekas grade 2–3 (deep) or grade 3 (periventricular), %	33.3
Presence of lacunes, %	70.8
PVS (at least 11), %	29.2
Presence of CMBs, %	43.8

### 3.1 Univariate analysis

The presence of lacunes was significantly correlated with a worse outcome measured by the binary mRS (*p* < 0.05, *X* = +5.215). Ethnicity (*p* < 0.05, *X* = +5.512), atrial fibrillation (*p* < 0.05, *X* = +8.926), categorical WMH (*p* < 0.05, *X* = +7.922) and WMH volume (*p* < 0.05, *t* = +2.167) correlated with the stroke etiology of large artery atherosclerosis. Atrial fibrillation (*p* < 0.05, *X* = +14.236) and race (*p* < 0.05, *t* = +2.093) correlated with cardioembolic stroke etiology. Both categorical WMH (*p* < 0.05, *X* = +5.538) and WMH volume (*p* < 0.05, *t* = −3.443) correlated with small vessel occlusion, as did the categorical SVD score (*p* < 0.05, *t* = −2.333). Smoking history correlated with stroke of other determined etiology (*p* < 0.05, *X* = +5.073). Heart failure was correlated with recurrent stroke (*p* < 0.05, *X* = +6.927). Diabetes mellitus (*p* < 0.05, *t* = +2.068), hyperlipidemia (*p* < 0.05, *t* = −2.518), and increased age (*p* < 0.05, *r* = +0.408) correlated with a higher NIHSS score. The presence of diabetes mellitus was associated with a longer ICU stay (*p* < 0.05, *t* = −2.786), whereas the presence of atrial fibrillation and number of CMBs was associated with a longer total length of stay (*p* = 0.05, *t* = −2.009 and *p* < 0.05, *r* = +0.362, respectively). After controlling for multiple comparisons using FDR correction, the following correlations with stroke etiologies remained statistically significant (*p* < 0.05): atrial fibrillation with cardioembolism, atrial fibrillation and categorical WMH with large artery atherosclerosis, and WMH volume with small vessel occlusion.

### 3.2 Multivariate analysis

**Figure 2:**
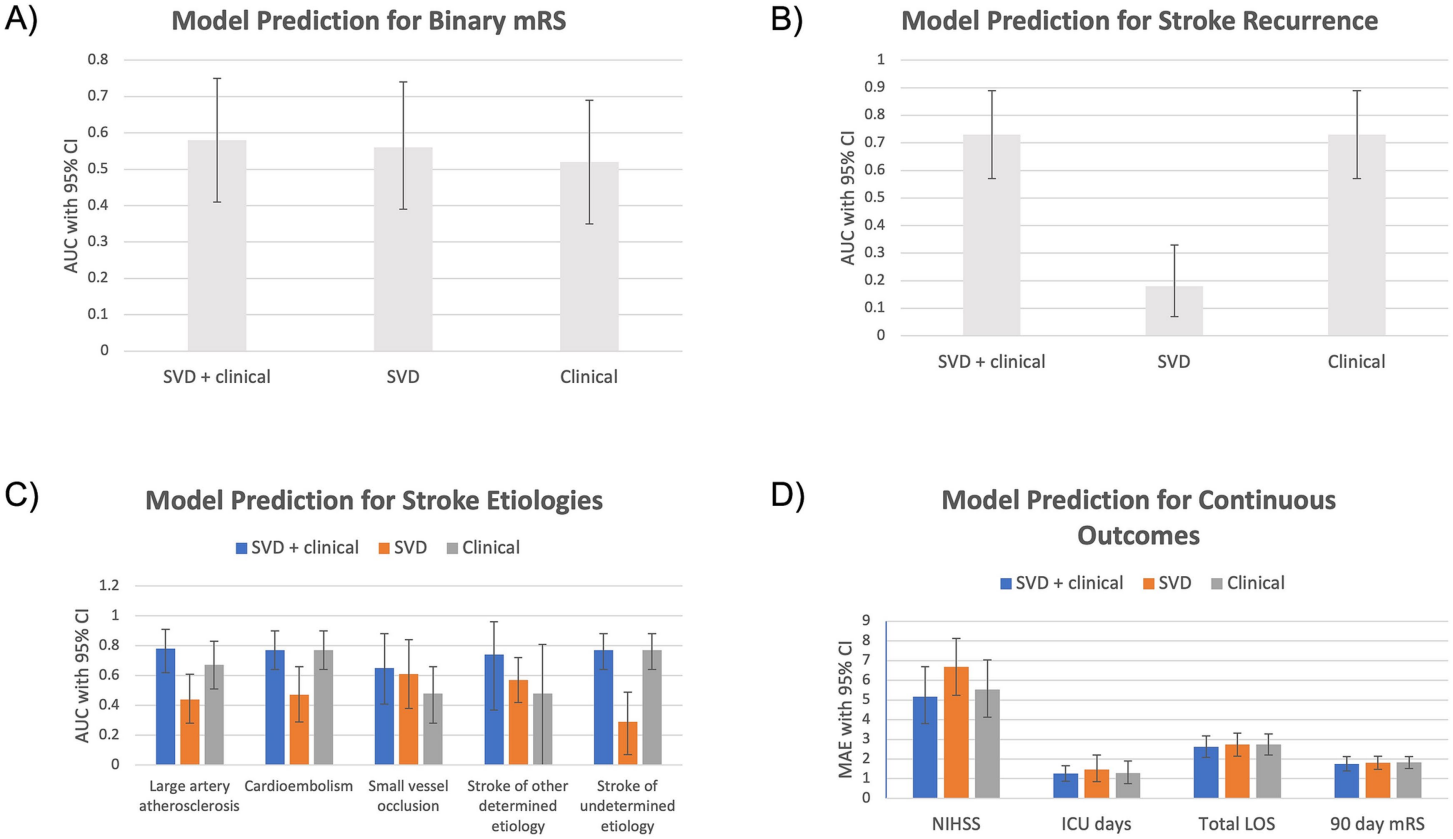
Bar graphs comparing model performance for prediction of stroke outcomes, including: **(A)** binary MRS, **(B)** stroke recurrence, **(C)** stroke etiologies and **(D)** NIHSS, ICU days, total LOS, and 90 day mRS.

Three models were evaluated in predicting stroke etiologies and outcomes: (1) SVD score, (2) clinical factors, and (3) combined SVD score + clinical factors. The models differed statistically significantly from each other across all outcome measures, based on pairwise comparisons using the Wilcoxon signed-rank test. Table 3 and Figure 2 compare overall model performance, and Supplemental Table 1 includes all other performance metrics.

**Table 3:** Comparison of SVD score, clinical factors, and combined SVD + clinical factors models to predict stroke outcomes.

**Multivariate analysis**	**SVD**	**Clinical**	**SVD + Clinical**
*Clinical outcomes*			
Binary mRS	0.56 (0.39–0.74)	0.52 (0.35–0.69)	0.58 (0.41–0.75)
NIHSS (MAE)	6.68 (5.23–8.12)	5.52 (4.13–7.03)	5.16 (3.80–6.69)
ICU days (MAE)	1.46 (0.85–2.20)	1.29 (0.74–1.91)	1.26 (0.86–1.66)
Total length of stay (MAE)	2.74 (2.14–3.32)	2.74 (2.21–3.27)	2.62 (2.08–3.17)
90 day mRS (MAE)	1.81 (1.48–2.15)	1.82 (1.52–2.13)	1.74 (1.39–2.12)
*Stroke etiology*			
Large artery atherosclerosis	0.44 (0.28–0.61)	0.67 (0.51–0.83)	0.78 (0.62–0.91)
Cardioembolism	0.47 (0.29–0.66)	0.77 (0.64–0.90)	0.77 (0.64–0.90)
Small vessel occlusion	0.61 (0.38–0.84)	0.48 (0.28–0.66)	0.65 (0.41–0.88)
Stroke of other determined etiology	0.57 (0.42–0.72)	0.48 (0–0.81)	0.74 (0.37–0.96)
Stroke of undetermined etiology	0.29 (0.07–0.49)	0.77 (0.64–0.88)	0.77 (0.64–0.88)
*Recurrent stroke*	0.18 (0.07–0.33)	0.73 (0.57–0.89)	0.73 (0.57–0.89)

Area under the ROC curve (AUC) is reported for categorical outcomes and mean absolute error (MAE) for continuous outcomes, with 95% confidence intervals.

#### 3.2.1 SVD score

The SVD score predicted the binary mRS with AUC 0.56 (95% CI: 0.39–0.74). The SVD score demonstrated a range of performance for predicting stroke etiologies with AUC ranges from 0.29 (0.07–0.49) for stroke of undetermined etiology to 0.61 (0.38–0.84) for small vessel occlusion. There was poor performance for recurrent stroke prediction with AUC 0.18 (0.07–0.33). For continuous outcomes, the SVD score predicted NIHSS with MAE 6.68 (5.23–8.12), ICU days MAE 1.46 (0.85–2.20), total length of stay MAE 2.74 (2.14–3.32), and 3-month mRS MAE 1.81 (1.48–2.15).

#### 3.2.2 Clinical factors

The clinical factors model predicted binary mRS with AUC 0.52 (0.35–0.69). Stroke etiology AUC ranged from AUC 0.48 for small vessel occlusion (95% CI: 0.28–0.66) and stroke of other determined etiology (95% CI: 0–0.81) to AUC 0.77 for cardioembolism (95% CI: 0.64–0.90) and stroke of undetermined etiology (95% CI: 0.64–0.88). Recurrent stroke AUC was 0.73 (0.57–0.89). Mean absolute error for NIHSS was 5.52 (4.13–7.03), ICU days 1.29 (0.74–1.91), total length of stay 2.74 (2.21–3.27), and 3-month mRS 1.82 (1.52–2.13).

#### 3.2.3 SVD score + clinical factors

The combined model predicted binarized mRS with AUC 0.58 (0.41–0.75). Stroke etiology prediction improved for large artery atherosclerosis 0.78 (0.62–0.91), small vessel occlusion 0.65 (0.41–0.88), and stroke of other determined etiology 0.74 (0.37–0.96). The other categorical outcomes, including cardioembolism and stroke of undetermined etiology, as well as recurrent stroke, did not improve with the combined model over the clinical model.

The combined model also lowered MAE for continuous outcomes: NIHSS MAE 5.16 (3.80–6.69), ICU days 1.26 (0.86–1.66), total length of stay 2.62 (2.08–3.17), and 3-month mRS 1.74 (1.39–2.12).

## 4 Discussion

The purpose of this study was to develop a semi-automated method of calculating an SVD score and evaluate its performance in predicting stroke outcomes. The univariate analysis found that the SVD score significantly correlated with small vessel occlusion etiology of stroke. Individual components of the SVD score also had significant associations including lacunes correlating with worse binary mRS and both categorical and continuous WMH correlating with large artery atherosclerosis etiology of stroke. WMH presence also positively correlated with small vessel occlusion, but when analyzing the volume of WMH, there was a negative correlation. Number of microbleed lesions correlated with length of total hospital stay. In multivariate analysis, the combined SVD + clinical factors model outperformed the other models in predicting functional independence and stroke etiologies of large artery atherosclerosis, small vessel occlusion, and stroke of other determined etiology. The combined model also demonstrated the lowest MAE across all continuous outcomes, including NIHSS, ICU days, total length of stay, and 90 day mRS.

Across nearly all outcomes, the combined SVD + clinical factors model outperformed the other two models, underscoring the added predictive value of a multimodal approach to modeling stroke outcomes and etiologies. Our results show that the SVD score provides some added value in terms of predicting stroke outcomes when combined with clinical factors. This finding is in agreement with previous literature which has demonstrated that higher SVD scores are associated with increased risk of recurrent ischemic stroke and worse post-stroke cognitive and functional outcomes (14, 19). Similar to prior studies, our model incorporated SVD severity in addition to the traditional SVD score to provide a more comprehensive assessment of SVD imaging markers in predicting stroke outcomes (13, 14). Small vessel occlusion discrimination was moderate for the combined and SVD alone models, but poor for the clinical factors model, highlighting the relevance of SVD imaging markers in identifying this stroke etiology. The combined model had the lowest MAE for predicting ICU days and total LOS, suggesting the utility of this model for resource allocation and risk stratification.

However, these results should be interpreted with caution given marginal improvement for some variables. For binary MRS, all models demonstrated fair to poor performance in predicting functional independence, suggesting that additional inputs are needed to improve prognostication. Cardioembolism and stroke of undetermined etiology were predicted equally well by the combined model and the clinical factors model, but not the SVD alone model. Thus, imaging biomarkers alone are likely not sufficient for classifying these stroke subtypes. The MAE for NIHSS was high across all models, suggesting that the three models may not be able to accurately estimate stroke severity and highlight the need for improved model calibration or incorporation of additional clinical inputs when attempting to predict NIHSS. Similarly, the models had moderate errors for 90 day mRS prediction and would need to be improved for individual prognostication.

Some of the marginal improvement could possibly be explained by the existing overlap of SVD score components with known vascular risk factors (10). Although prior literature has found that an SVD score greater than or equal to two is by itself associated with increased risk for recurrent stroke after adjustment for other risk factors, measurement of the SVD score has not been optimized, with different studies incorporating different metrics (13, 14, 20).

While this initial SVD analysis shows promise in aiding stroke prognostication, there are several limitations to this study. The modest sample size and retrospective design at a single institution limits generalizability. Nearly all patients in our cohort had hypertension (47/48) which prevented the incorporation of this important vascular risk factor into the statistical models. This exclusion limits the model’s capacity to evaluate interactions between hypertension and SVD imaging burden. For imaging analysis, we used different MRI vendors, field strengths, and imaging parameters which could contribute to variability in imaging interpretation. In addition, bias field correction was not done to account for field inhomogeneities of the various scanners, which may limit interpretation of subtle features. Evaluation for stroke recurrence was only assessed in the time frame of the study, and no long-term longitudinal follow-up was performed. Also notably, SVD markers like WMH and CMBs are highly prevalent in older adults and may be influenced by non-stroke conditions such as chronic hypertension, cerebral amyloid angiopathy, and age-related microangiopathy (21). These overlapping pathologies could bias associations with stroke outcomes, particularly in older populations where distinguishing causal from incidental findings is challenging. This may explain the apparent contradiction we found in categorical WMH being positively associated with small vessel occlusion while WMH volume was negatively correlated, possibly reflecting threshold effects in categorical classification versus the broader vascular burden captured by volumetric quantification. Such discrepancies underscore the importance of measurement method and the heterogeneity of underlying cerebrovascular pathology. Future models may benefit from incorporating the location of lesions to help disambiguate these contributions, as it has been shown that hypertensive arteriopathy and cerebral amyloid angiopathy may result in different WMH distribution patterns, but this will require further exploration. (21) Despite these shortcomings, our results corroborate findings in previous studies that use larger sample sizes, and the combined SVD and clinical factors model consistently had better predictive value for nearly all outcomes (11, 14, 19).

Future studies could utilize a larger sample size to assess the generalizability of our model. Optimization of the model using various thresholds could also be tested. While our combined SVD + clinical factors model yielded statistically significant improvements in predicting nearly all outcomes, the clinical relevance of this improvement is unknown. Real-world impact may depend on whether these improvements meaningfully influence triage, therapy decisions, or patient counseling, and future validation in larger prospective cohorts is needed.

This tool may have value in specific clinical workflows. For instance, early identification of high SVD burden could aid in triage decisions in acute stroke units, helping clinicians predict which patients are more likely to experience poor outcomes despite reperfusion therapy. Additionally, SVD-informed stratification could guide secondary prevention strategies, identifying patients at elevated risk for recurrence who may benefit from closer monitoring, aggressive risk factor modification, or targeted rehabilitation planning. Integration into electronic health record systems or stroke decision support tools could further streamline its use in real time.

Although this study used a semi-automated method to calculate an SVD score, we envision a fully automated deep learning AI method in the future which integrates automated segmentation of SVD features and a predictive model capable of incorporating imaging features and clinical data into outcome prediction. Benchmarking against existing open-source or commercial solutions for SVD feature segmentation could provide critical evaluation for relative performance and improvements to generalizability.

In sum, we evaluated a semi-automated SVD score to predict stroke outcomes, with the incorporation of SVD score to clinical factors allowing for better predictive value than either alone.

## Data Availability

The raw data supporting the conclusions of this article will be made available by the authors, without undue reservation.
