# Supplementary material for: Evaluation of small vessel disease burden on MRI and stroke outcomes
Source: Front Neurol. 2025 Jul 9;16:1628787. doi: 10.3389/fneur.2025.1628787 (PMC12285601; doi:10.3389/fneur.2025.1628787)
Supplement: Supplementary file 1 [file Table_1.DOCX]

**Supplemental Table 1:** Comparison of the three models’ performance metrics for these categorical outcomes: binary mRS (1a), large artery atherosclerosis (1b), cardioembolism (1c), small vessel occlusion (1d), stroke of other determined etiology (1e), stroke of undetermined etiology (1f), and recurrent stroke (1g).

(1a)

| **Outcome: Binary mRS** | **SVD** | **Clinical** | **SVD + Clinical** |
| --- | --- | --- | --- |
| Accuracy | 0.69 | 0.60 | 0.71 |
| Sensitivity | 0.41 | 0.82 | 0.36 |
| Specificity | 0.92 | 0.42 | 1.00 |
| Positive predictive value | 0.82 | 0.55 | 1.00 |
| Negative predictive value | 0.65 | 0.73 | 0.65 |

(1b)

| **Outcome: Large artery atherosclerosis** | **SVD** | **Clinical** | **SVD + Clinical** |
| --- | --- | --- | --- |
| Accuracy | 0.63 | 0.65 | 0.77 |
| Sensitivity | 0.94 | 0.94 | 0.88 |
| Specificity | 0.47 | 0.50 | 0.72 |
| Positive predictive value | 0.47 | 0.48 | 0.61 |
| Negative predictive value | 0.94 | 0.94 | 0.92 |

(1c)

| **Outcome: Cardioembolism** | **SVD** | **Clinical** | **SVD + Clinical** |
| --- | --- | --- | --- |
| Accuracy | 0.63 | 0.77 | 0.77 |
| Sensitivity | 0.71 | 0.76 | 0.76 |
| Specificity | 0.58 | 0.77 | 0.77 |
| Positive predictive value | 0.48 | 0.65 | 0.65 |
| Negative predictive value | 0.78 | 0.86 | 0.86 |

(1d)

| **Outcome: Small vessel occlusion** | **SVD** | **Clinical** | **SVD + Clinical** |
| --- | --- | --- | --- |
| Accuracy | 0.71 | 0.46 | 0.81 |
| Sensitivity | 0.67 | 0.89 | 0.67 |
| Specificity | 0.72 | 0.36 | 0.85 |
| Positive predictive value | 0.35 | 0.24 | 0.50 |
| Negative predictive value | 0.90 | 0.93 | 0.92 |

(1e)

| **Outcome: Stroke of other determined etiology** | **SVD** | **Clinical** | **SVD + Clinical** |
| --- | --- | --- | --- |
| Accuracy | 0.60 | 0.75 | 0.88 |
| Sensitivity | 1.00 | 0.50 | 0.75 |
| Specificity | 0.57 | 0.77 | 0.89 |
| Positive predictive value | 0.17 | 0.17 | 0.38 |
| Negative predictive value | 1.00 | 0.94 | 0.98 |

(1f)

| **Outcome: Stroke of undetermined etiology** | **SVD** | **Clinical** | **SVD + Clinical** |
| --- | --- | --- | --- |
| Accuracy | 0.42 | 0.79 | 0.79 |
| Sensitivity | 0.75 | 1.00 | 1.00 |
| Specificity | 0.39 | 0.77 | 0.77 |
| Positive predictive value | 0.10 | 0.29 | 0.29 |
| Negative predictive value | 0.94 | 1.00 | 1.00 |

(1g)

| **Outcome: Recurrent stroke** | **SVD** | **Clinical** | **SVD + Clinical** |
| --- | --- | --- | --- |
| Accuracy | 0.77 | 0.69 | 0.69 |
| Sensitivity | 0.00 | 0.80 | 0.80 |
| Specificity | 0.97 | 0.66 | 0.66 |
| Positive predictive value | 0.00 | 0.38 | 0.38 |
| Negative predictive value | 0.79 | 0.93 | 0.93 |
